# *Porin* Expression Profiles in *Haemaphysalis longicornis* Infected With *Babesia microti*

**DOI:** 10.3389/fphys.2020.00502

**Published:** 2020-05-19

**Authors:** Weiqing Zheng, Rika Umemiya-Shirafuji, Qian Zhang, Kiyoshi Okado, Paul Franck Adjou Moumouni, Hiroshi Suzuki, Haiying Chen, Mingming Liu, Xuenan Xuan

**Affiliations:** ^1^National Research Center for Protozoan Diseases, Obihiro University of Agriculture and Veterinary Medicine, Obihiro, Japan; ^2^The Collaboration Unit for Field Epidemiology of State Key Laboratory for Infectious Disease Prevention and Control, Jiangxi Provincial Key Laboratory of Animal-origin and Vector-Borne Diseases, Nanchang Center for Disease Control and Prevention, Nanchang, China; ^3^The Ophthalmology Department, The Second Affiliated Hospital of Nanchang University, Nanchang, China

**Keywords:** *Haemaphysalis longicornis*, tick, *Babesia microti*, protozoan parasite, *porin*, expression profiles

## Abstract

The *porin* gene is widely disseminated in various organisms and has a pivotal role in the regulation of pathogen infection in blood-sucking arthropods. However, to date, information on the *porin* gene from the *Haemaphysalis longicornis* tick, an important vector of human and animal diseases, remains unknown. In this study, we identified the *porin* gene from *H. longicornis* and evaluated its expression levels in *Babesia microti*-infected and -uninfected *H. longicornis* ticks at developmental stages. We also analyzed *porin* functions in relation to both tick blood feeding and *Babesia* infection and the relationship between *porin* and *porin*-related apoptosis genes such as *B-cell lymphoma* (*Bcl*), *cytochrome complex* (*Cytc*), *caspase 2* (*Cas2*), and *caspase 8* (*Cas8*). The coding nucleotide sequence of *H. longicornis porin* cDNA was found to be 849 bp in length and encoded 282 amino acids. Domain analysis showed the protein to contain six determinants of voltage gating and two polypeptide binding sites. *Porin* mRNA levels were not significantly different between 1-day-laid and 7-day-laid eggs. In the nymphal stage, higher *porin* expression levels were found in unfed, 12-h-partially-fed (12 hPF), 1-day-partially-fed (1 dPF), 2 dPF nymphs and nymphs at 0 day post-engorgement (0 dAE) vs. nymphs at 2 dAE. *Cytc* and *Cas2* mRNA levels were higher in 2 dPF nymphs in contrast to nymphs at 2 dAE. *Porin* expression levels appeared to be higher in the infected vs. uninfected nymphs during blood feeding except at 1 dPF and 0–1 dAE. Especially, the highest *B. microti* burden negatively affected *porin* mRNA levels in both nymphs and female adults. *Porin* knockdown affected body weight and *Babesia* infection levels and significantly downregulated the expression levels of *Cytc* and *Bcl* in *H. longicornis* female ticks. In addition, this study showed that infection levels of the *B. microti* Gray strain in nymphal and female *H. longicornis* peaked at or around engorgement from blood feeding to post engorgement. Taken together, the research conducted in this study suggests that *H. longicornis porin* might interfere with blood feeding and *B. microti* infection.

## Introduction

The Asian longhorned tick, *Haemaphysalis longicornis*, is widely distributed in eastern Asia, Australia, and New Zealand and was recently found in the US (Heath, 2016; Rainey et al., 2018; Raghavan et al., 2019; Wormser et al., 2019; Zheng et al., 2019). *H. longicornis*, known as a harmful ectoparasite for domestic animals, spreads diseases including babesioses to livestock (McFadden et al., 2011). The tick has also been associated with several other tick-borne diseases in humans, including bacterioses and viroses (Chae and Lee, 2010; Fang et al., 2015; Zheng et al., 2018; Zhuang et al., 2018).

Over millions of years, ticks have co-evolved with a variety of microorganisms including *Babesia*. When *Babesia* parasites enter the tick body, ticks activate their immune system to inhibit *Babesia* invasion, and in turn, *Babesia* parasites hijack various tick molecules to facilitate their own transmission (de la Fuente et al., 2017). Several molecules are essential for tick-*Babesia* interaction, such as defensins, microplusin/hebraein, Kunitz domain-containing proteins, lipocalins, and proteases (Antunes et al., 2017). It is speculated that *porin*, also termed a voltage-dependent anion-selective channel (VDAC), plays paramount roles in modulating pathogen infection in vectors, including bacteria and protozoa in ticks, and viruses in mosquitoes (Fongsaran et al., 2014; Alberdi et al., 2015; Rodríguez-Hernández et al., 2015; Jitobaom et al., 2016). To date, *porin* has been described in at least three tick species, including *Ixodes scapulari*s, *Rhipicephalus microplus*, and *Amblyomma variegatum* (Ribeiro et al., 2011; Rodríguez-Hernández et al., 2011; Alberdi et al., 2015). *Porin* in *R. microplus* was identified when it was exposed to *Babesia bigemina* infection (Rodríguez-Hernández et al., 2011).

Various *Babesia* parasites including *Babesia microti* have been experimentally transmitted by or detected in the Asian longhorned tick (Ikadai et al., 2007; Sivakumar et al., 2014; Fang et al., 2015; Zhang et al., 2017). *B. microti* is the most malignant human *Babesia* parasite with high morbidity and wide distribution around the globe (Vannier and Krause, 2012; Chen et al., 2019; Krause, 2019), and *Ixodes* ticks have historically been considered as common vectors of *B. microti* (Mather et al., 1990; Krause, 2019). However, *B. microti* DNA can be detected in *H. longicornis* collected from the field (Zhang et al., 2017) and can be acquired by the tick when feeding on mice infected with the *B. microti* Munich strain (Kusakisako et al., 2015). The transmission of *B. microti* from *H. longicornis* to mice has also been achieved (Wu et al., 2017), suggesting that the tick is a potential vector of the protozoan parasite. However, the molecular mechanisms underlying *H. longicornis-B. microti* interactions remain unclear.

On the basis of the above information, we hypothesized that *H. longicornis porin* might have roles in modulating *B. microti* infection in the ticks, and thus we designed experiments to confirm the hypothesis in this study. First, a homolog of *porin* was identified and characterized in *H. longicornis* using an Expressed Sequence Tags (ESTs) database, and then the expression levels of *porin* mRNA in *H. longicornis* eggs and nymphs were analyzed by real-time PCR. Moreover, we established a *H. longicornis*-*B. microti* Gray strain (a human-pathogenic strain) infection model and determined the dynamics of *B. microti* loads in nymphal and female ticks during the blood feeding stage. *Porin* mRNA transcripts were then compared between *B. microti*-infected and -uninfected ticks. Finally, *porin* functional analyses were carried out by RNA interference (RNAi) to determine its potential roles in blood feeding and *B. microti* infection.

## Materials and Methods

### Ticks, Parasites, and Animals

Parthenogenetic *H. longicornis* ticks (Okayama strain) were kept at the National Research Center for Protozoan Diseases (NRCPD), Obihiro University of Agriculture and Veterinary Medicine, Obihiro, Japan, and maintained by feeding on the ears of Japanese white rabbits (Japan SLC, Shizuoka, Japan) (Umemiya-Shirafuji et al., 2019a). In the present study, two rabbits were used to maintain the nymphal and female ticks. The *B. microti* Gray strain was used to produce *B. microti*-infected ticks. Cryopreserved protozoan parasites were kept in liquid nitrogen in NRCPD and thawed using the methods mentioned in The Global Bioresource Center (ATCC^®^ 30221^TM^). Seven 8-week-old female hamsters (Japan SLC, Shizuoka, Japan) were inoculated with thawed *B. microti* and then used for blood feeding to produce *B. microti*-infected ticks. In parallel, seven uninfected hamsters were used for blood meal and production of uninfected ticks. All animals used in this study were reared in a temperature- and humidity-regulated room under controlled lighting, given water and commercial regular chow, and were cared for in accordance with the guidelines approved by the Animal Care and Use Committee (Animal exp.: 19–74 for rabbits and 19–77 for hamsters) of Obihiro University of Agriculture and Veterinary Medicine.

### Identification and Characterization of the cDNA Encoding *Porin*

ESTs were previously constructed by random partial sequencing of the 5′- terminal of the cDNA clones from cDNA libraries established with salivary glands of 4-day-fed *H. longicornis* females, and the similarities in the protein databases were examined using the BLASTp program (Liao et al., 2009). The plasmids containing the *porin* gene-encoding insert were extracted using a Qiagen DNA purification kit (Qiagen, Hilden, Germany) and subsequently subjected to analysis on an ABI PRISM 3100 DNA sequencer (Applied Biosystems, Waltham, MA, United States) using plasmid (pGCAP1 vector)-specific primers and walking primers thereafter.

The full length of the *porin* coding region was searched with the BLASTx program in the National Center for Biotechnology Information (NCBI)^1^. The domain structure was determined using the Conserved Protein Domain Family search program in the NCBI^2^. The deduced amino acid translation of the *porin* sequence was performed using an online tool Nucleotide Amino acid Derived Visualization^3^. Alignment of the *porin* amino acid sequences from different tick species was viewed with the Multiple Align Show^4^. The identity and similarity between *H. longicornis* and other tick species were calculated with the Ident and Sim program of the Sequence Manipulation Suite. The similar amino acids were classified into the same group for the similarity calculation: GAVLI, FYW, CM, ST, KRH, DENQ, and P^5^. The theoretical pI (isoelectric point) and Mw (molecular weight) were determined by the Compute pI/Mw^6^.

### Real-Time PCR Analysis

The expression levels of the *porin* gene were analyzed in ticks at egg, nymph, or adult stage, in ticks incubated at 15°C or 25°C, and in *B. microti*-infected or -uninfected ticks. Three duplicates were made for each group of tick samples. After two washes with double distilled water and one wash with 70% ethanol, 10 mg of eggs, whole body of four nymphs, and three unfed and two partially fed or engorged female ticks with host blood removed were homogenized in TRI reagent (Sigma-Aldrich, St. Louis, MO, United States) using pestles. RNA extraction, cDNA synthesis, and real-time PCR were performed as described elsewhere (Umemiya-Shirafuji et al., 2019b). The same amount of cDNA was used in a real-time PCR reaction system to assess the stability of internal control genes in ticks at different developmental stages under unfed, uninfected, or infected conditions. The candidate internal control genes evaluated in this study included glyceraldehyde-3-phosphate dehydrogenase (*GAPDH*), *L23*, *HlP0*, and *Hlactin*. The most stable one was used for analysis of the relative mRNA level of the *porin* gene. *Porin*-related apoptosis genes such as *B-cell lymphoma* (*Bcl*), *cytochrome complex* (*Cytc*), *caspase 2* (*Cas2*), and *caspase 8* (*Cas8*) were also assessed by real-time PCR. The *H. longicornis Bcl* sequence was identified using the EST database as described above, and for the other genes, previously published sequences were used (GenBank database under accession number DQ666174 for *Cas2*, DQ660369 for *Cas8*, and NC_037493 for *Cytc*). The primers used in our study are listed in Supplementary Table S1. The mRNA levels were normalized separately against mRNA levels of the internal control gene using the ΔCT {2^^[–^^(CT^_target gene_^–CT^_internal control gene_^)]^} method.

### Analyses of *B. microti* Burdens in Ticks

*B. microti* burdens were calculated in nymphal and female ticks by standardizing the relative amount of *Babesia* 18S rRNA against tick ITS-2 in infected ticks with the values obtained in uninfected ones. The amounts of *Babesia* 18S rRNA and tick ITS-2 in the samples were evaluated using genomic DNA samples for real-time PCR, and the practice was repeated thrice for each group. Tick samples consisted of nymphs, which were allowed to feed on *B. microti*-infected hamsters with ∼10% parasitemia, and female ticks, which fed on hamsters with ∼5% parasitemia. Genomic DNA was isolated from *B. microti*-infected ticks using a NucleoSpin^®^ Tissue kit (Macherey-Nagel, Duren, Germany) according to the manufacturer’s manual. In addition, conventional PCR using KOD-Plus-Neo DNA polymerase (Toyobo, Osaka, Japan) and *B. microti β-tubulin*-specific primers and *H. longicornis actin*-specific primers (control gene) was performed on nymphal samples to detect *B. microti* DNA. The PCR products were electrophoresed on a 1.5% agarose gel and stained with ethidium bromide (EB). Conventional PCR was performed in triplicate for each group. The primers used in this study are listed in Supplementary Table S1. The genetic amount of *B. microti* 18S rRNA (Bm18S rRNA) was normalized against that of *H. longicornis* ITS-2 (HlITS-2) using the ΔCT {2^^[–(CT _Bm18S rRNA_ –CT _HlITS–2_)]^} method.

### Suppression Subtractive Hybridization (SSH) cDNA Construction and Analysis

The technique of SSH was used to compare the expression levels of the *porin* gene in *B. microti*-infected and -uninfected engorged female ticks. *Babesia* DNA in ticks was detected by conventional PCR with *β-tubulin* gene primers as described above. Forward and reverse suppression subtraction cDNA libraries were constructed using the Super SMART^TM^ PCR cDNA synthesis kit according to the manufacturer’s instructions (Clontech, Mountain View, CA, United States). Briefly, in the forward suppression subtraction cDNA library, cDNA prepared from 15 *Babesia*-infected ticks served as the “Tester,” and cDNA prepared from 15 uninfected ticks served as the “Driver” in the subtraction procedure to enrich for cDNAs preferentially expressed and upregulated in the *Babesia*-infected ticks. In the reverse suppression subtraction cDNA library, cDNA from 15 infected ticks (driver) was used in excess to hybridize cDNA from 15 uninfected ticks (tester) to enrich for cDNAs preferentially expressed and upregulated in the uninfected ticks. Two PCR amplifications were performed to enrich differentially expressed transcripts in infected ticks from the forward suppression subtraction cDNA library and in uninfected ticks from the reverse suppression subtraction cDNA library. The amounts of *porin* transcripts in the forward and reverse suppression subtraction cDNA libraries and in unsubtracted cDNA libraries were determined by relative band brightness of its PCR products on an electrophoresed gel stained with EB.

### RNAi and the Effect of *Porin* Knockdown on Tick Blood Feeding and *Babesia* Infection

RNAi was used to analyze the effect of *porin* knockdown on blood feeding, *Babesia* infection, and the *porin*-related apoptosis signaling pathway. The *porin* double-strand RNA (dsRNA) was constructed with the primer set including the T7 promoter sequence (underlined with double solid lines) at the 5′-end of both primers (*porin* RNAiF: 5′-GATA TCTAATACGACTCACTATAGGTGCACACCAACGTGAACG AC-3′; *porin* RNAiR: 5′-GATATCTAATACGACTCACTATAGG AAAAGATAGGAAGGGTCTGCCG-3′). Female ticks were used for RNAi experiments as described previously (Liao et al., 2009). The dsRNA-injected ticks were allowed to rest 1 day and then put in chambers attached to the hair-shaved back of hamsters. Each hamster was challenged with 15 dsRNA-injected ticks in the control group or experimental group. The practice was repeated three times. To examine *porin* knockdown efficiency during blood feeding after dsRNA injection, two 0-to-7-day-fed ticks from the infested hamsters were collected from the *porin* dsRNA-injected group and a firefly *luciferase* dsRNA-injected group as a control. Determination of the expression of *porin* was done as described in Section Real-Time PCR Analysis. In contrast, *B. microti* burdens and the expression levels of *porin*-related apoptosis genes were assessed by real-time PCR using genomic DNA and cDNA, respectively. The feeding success of the remaining ticks was investigated by measuring the feeding period and body weight at engorgement.

### Statistical Analysis

The mean ranks of *Babesia* burdens in the *porin* RNAi or control group and mRNA levels of *porin* or its related apoptosis genes in the uninfected or infected ticks, *porin* RNAi or control group, 1-day-laid or 7-day-laid eggs, and 2-day-partially-fed (2 dPF) or 2 days after engorgement (2 dAE) nymphs were compared using the Mann-Whitney *U* test. The difference in the mean ranks of *B. microti* burdens, and mRNA levels of *porin* in nymphal and female adult ticks during the blood-feeding process, was analyzed with the Kruskal-Wallis *H* test followed by the Dunn’s multiple comparisons test. A *p*-value of <0.05 was considered statistically significant.

### Nucleotide Sequence Accession Number

The sequences of the *porin* gene of *H. longicornis* and its related apoptosis gene *Bcl* were submitted to the GenBank database under accession numbers MN584740 and MN584741, respectively.

## Results

### *Porin* Characterization

**FIGURE 1:**
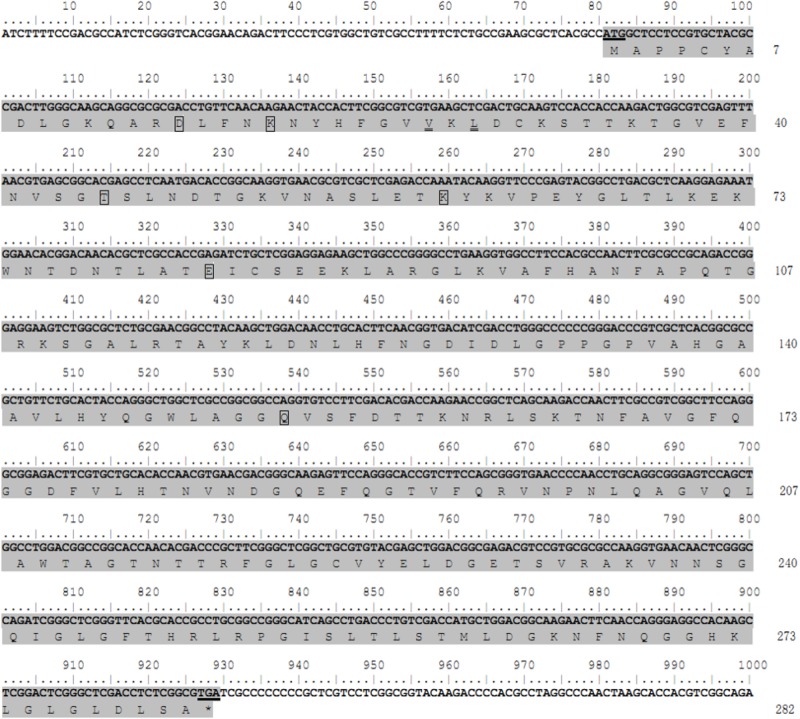
Nucleotide and deduced amino acid sequences of the *porin* gene from the *H. longicornis* tick. The protein coding region for the *porin* gene is indicated by gray shading. The residuals in the boxes show putative determinants of voltage gating, and the residuals with double underlines are putative polypeptide binding sites. The start codon ATG and stop codon TGA are underlined. Nucleotides are numbered above each line, and amino acid numbering is on the right.

**FIGURE 2:**
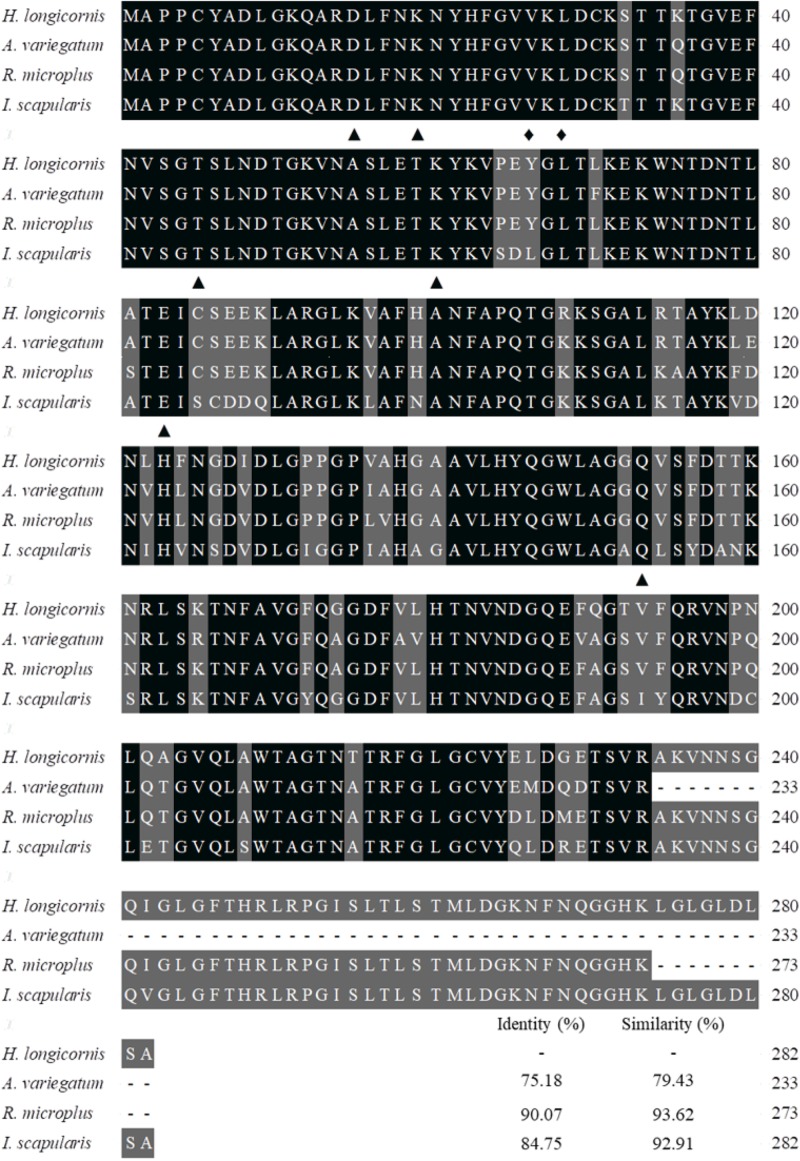
Alignment of the amino acid sequence of the *porin* gene of *H. longicornis* was compared with those of the ixodid ticks *Ixodes scapulari*s (XP_002408065), *Rhipicephalus microplus* (ADT82652), and *Amblyomma variegatum* (DAA34069). Identical residues are darkly shaded and similarity residues are gray shaded. Amino acid numbering is on the right. The putative determinants of voltage gating and polypeptide binding sites are shown at the bottom of the sub-columns with triangles and diamonds, respectively.

The coding nucleotide sequence of the *porin* cDNA was found to be 849 bp in length and encoded 282 amino acids with an expected isoelectric point of 8.95 and molecular weight of 30.4 kDa. The protein is glycine-and-leucine rich with 35 glycines and 31 leucines. Domain analysis showed *porin* to contain six determinants of voltage gating and two polypeptide binding sites (Figure 1). Multiple alignment of the amino acid sequence with the homolog sequences from other tick species, including *I. scapulari*s, *R. microplus*, and *A. variegatum*, revealed that the determinants of voltage gating and the polypeptide binding sites are conserved among these four tick species. The *H. longicornis porin* amino acids showed the highest homology with that of *R. microplus*, with 90.07% identity and 93.62% similarity, in contrast to 84.75% identity and 92.91% similarity with that of *I. scapularis*, and 75.18% identity and 79.43% similarity with that of *A. variegatum* (Figure 2).

### Expression Profiles of *Porin* Gene and *Porin*-Related Apoptosis Genes in *H. longicornis* Ticks

**FIGURE 3:**
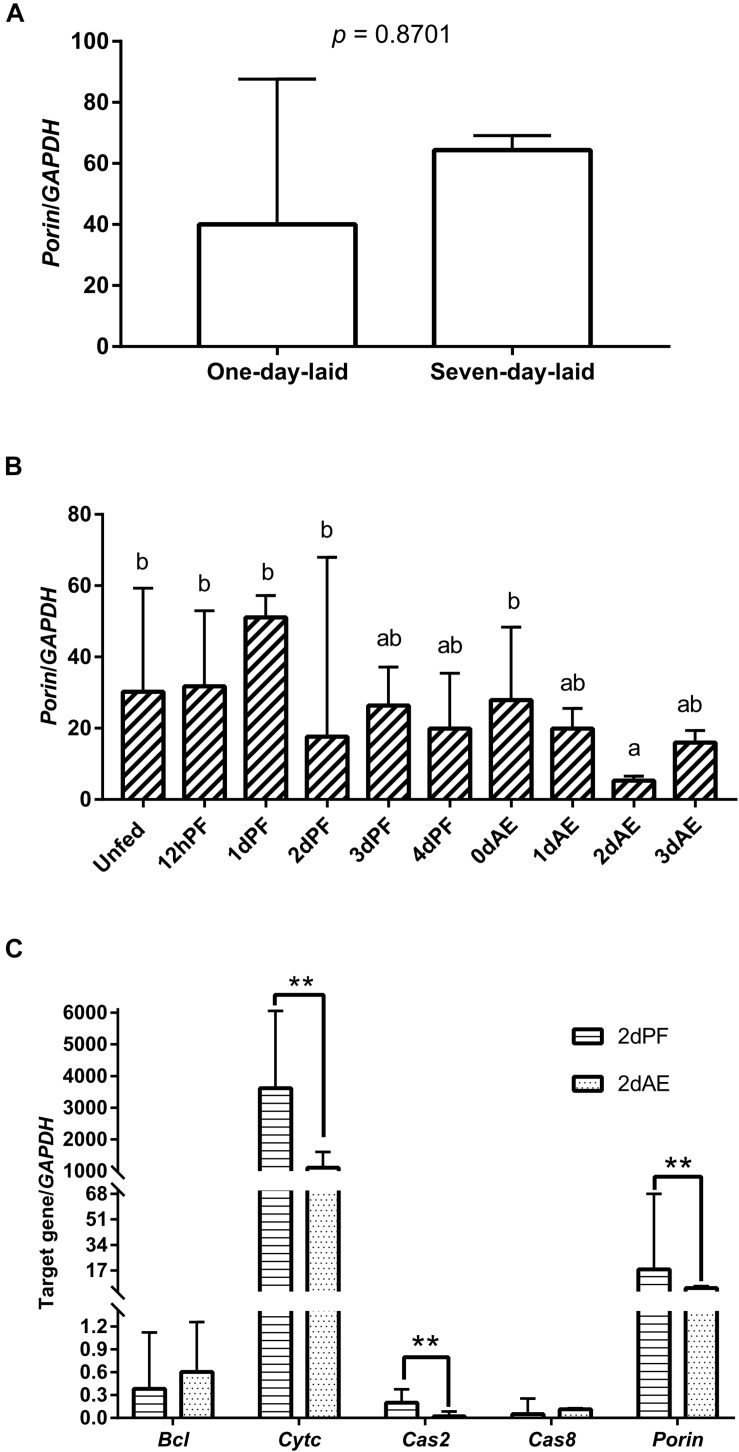
*Porin* expression levels at developmental stages of *H. longicornis* ticks. **(A)** *Porin* gene expression levels at egg stage (1-day-laid eggs and 7-day-laid eggs). **(B)** *Porin* gene expression levels in unfed nymphs, partially fed nymphs (12 hPF to 4 dPF), and nymphs at 0–3 days after engorgement (0 dAE to 3 dAE). **(C)** Comparison of mRNA expression levels of *porin* and its caspase-related apoptosis signal pathway genes between 2 dAE nymphs and 2 dPF nymphs. The bar indicates the median with 95% confidence interval (CI) of three biological repeats. Different letters above the bars represent significant differences (*p* < 0.05). **p < 0.01.

*GAPDH* was the most stably expressed internal control gene in ticks at developmental stages compared with *L23*, *HlP0*, and *Hlactin* and was used as the internal control gene in this study (Supplementary Figure S1). Real-time PCR revealed that *porin* mRNA was expressed in the eggs and unfed and fed nymphs (Figures 3A,B). There were no differences in *porin* mRNA levels between 1-day-laid eggs and 7-day-laid eggs (Figure 3A). *Porin* showed no significant change in expression levels between unfed nymphs incubated at 15 and 25°C (Supplementary Figure S2). *Porin* expression levels were higher in the unfed nymphs, 12-h-partially-fed (12 hPF) to 2-d-partially-fed (2 dPF) nymphs, and the nymphs at 0 dAE than the nymphs at 2 dAE (*p* < 0.05) (Figure 3B). Subsequently, nymphal samples at 2 dPF and 2 dAE were used to examine expression levels of *porin*-related apoptosis genes. *Cytc* and *Cas2* were significantly less expressed in 2 dAE nymphs than in 2 dPF nymphs (*p* < 0.05) (Figure 3C). However, mRNA levels of *Bcl* and *Cas8* in nymphs were not significantly different at 2 dAE vs. 2 dPF (Figure 3C).

### *B. microti* Gray Strain Burdens in *H. longicornis* Ticks

**FIGURE 4:**
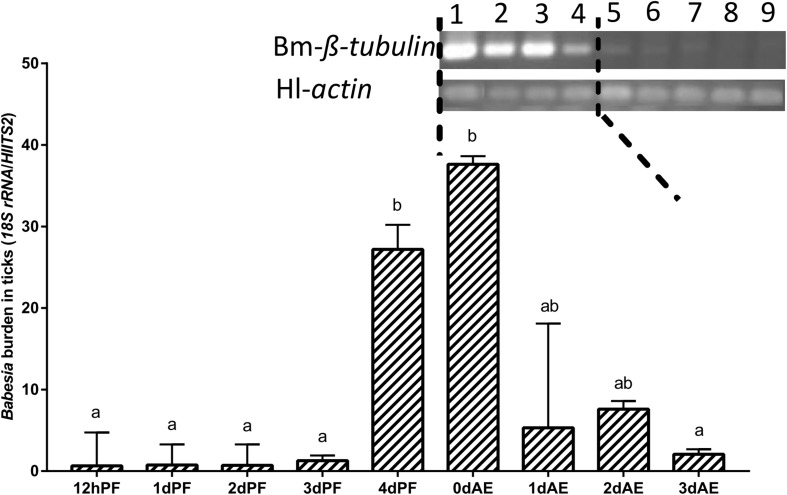
*Babesia* burdens in nymphal *H. longicornis* ticks. Gel electrophoresis image shows the result of a conventional PCR analysis of *Babesia* infection in nymphal ticks at 0–7 days after engorgement (lanes 1–8). Lane 9, negative control. β-*tubulin* (1,341 bp; Bm-β-*tubulin*) was amplified to detect *Babesia microti*, and a 143-bp fragment of the *H. longicornis actin* was amplified as a control. Data sets plotted in histogram are for 12-h partially fed nymphs to 4-day partially-fed nymphs (12 hPF to 4 dPF) and engorged nymphs at 0–3 days after engorgement (0 dAE to 3 dAE). The bar indicates the median with 95% CI of three biological repeats, and different letters above the bars represent significant differences (*p* < 0.05).

**FIGURE 5:**
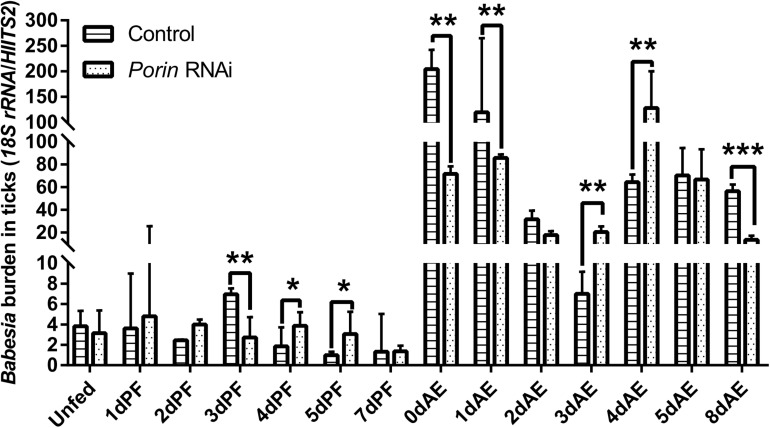
*Babesia* burdens in *porin* RNAi and control ticks during blood feeding. The bar indicates the median with 95% CI of three biological repeats. The asterisks above the bars indicate significant differences in *Babesia* burdens between *porin* RNAi and control groups. *p < 0.05; **p < 0.01; ***p < 0.001.

Nymphs fed on *B. microti*-infected hamsters for 12 h and 1–3 days (12 hPF nymphs to 3 dPF nymphs) had lower levels of *Babesia* burdens compared with those fed for 4 days (4 dPF nymphs) and ticks at the onset of engorgement (0 dAE) (Figure 4). Real-time PCR analysis showed that the largest amount of *Babesia* DNA was detected at 0 dAE and then decreased at 1–3 dAE, which was further confirmed by conventional PCR analysis (gel electrophoresis image in Figure 4). A similar phenomenon was found in female ticks injected with dsRNA of firefly *luciferase* (control group) during blood feeding as evidenced by the peak of *Babesia* burden at 0 dAE and its subsequent reduction (Figure 5).

### Comparison of Expression Levels of *Porin* Gene Between Uninfected and Infected Nymphal and Female Ticks

**FIGURE 6:**
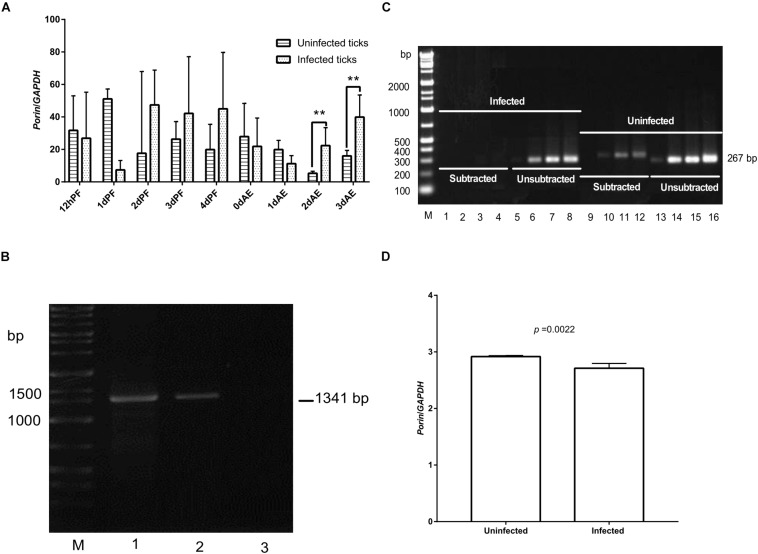
Expression levels of the *porin* gene between uninfected and infected nymphal and female ticks. **(A)** *Porin* gene expression level in *B. microti*-infected or -uninfected nymphal *H. longicornis*. **(B)** Gel electrophoresis analysis of conventional PCR products of *Babesia* β-*tubulin* (1,341 bp) in *B. microti*-infected and -uninfected female ticks at engorgement. Lane 1, infected tick sample; lane 2, positive control; lane 3, uninfected tick sample. **(C)** SSH analysis of *porin* expression levels in infected ticks. No or weak bands were visualized on a gel for PCR products of *porin* amplified from cDNA of infected ticks subtracted with that of uninfected ticks. Bright bands were visualized on a gel for PCR products of *porin* amplified from cDNA of uninfected ticks subtracted with that of infected ticks. Lanes 1, 5, 9, and 13: PCR products amplified by 18 cycles from *porin* gene; lanes 2, 6, 10, and 14: PCR products amplified by 23 cycles; lanes 3, 7, 11, and 15: PCR products amplified by 28 cycles; lanes 4, 8, 12, and 16: PCR products amplified by 33 cycles. **(D)** Real-time PCR analysis of *porin* mRNA expression in whole body of *B. microti*-infected and -uninfected engorged female ticks. The bar indicates the median with 95% CI of three biological repeats. The asterisks above the bar indicate a significant difference in *porin*/*GAPDH* between uninfected and *B. microti*-infected ticks (**p < 0.01).

Expression of the *porin* gene was found in *B. microti*-infected or -uninfected nymphs (Figure 6A). *Porin* expression levels were higher in the infected vs. uninfected nymphs at 2 and 3 dAE (Figure 6A). The expression levels appeared to be higher in infected nymphs during blood feeding (2, 3, and 4 dPF) compared with uninfected nymphs. When the highest *Babesia* load was reached at 0 dAE (Figure 4), it appeared that the *porin* expression level in the infected nymphs was decreased (*p = * 0.43) (Figure 6A). We then performed an experiment to validate whether the identical phenomenon occurred in female ticks (Figures 6B–D). The band on a gel in *porin* PCR products amplified from the reverse SSH cDNA library was brighter than those from the forward SSH cDNA library (Figure 6C). The significantly higher mRNA levels of *porin* in uninfected engorged female ticks were further confirmed by real-time PCR (*p* = 0.0022) (Figure 6D).

### Effect of *Porin* Knockdown on Blood Feeding, *Babesia* Infection, and Expression Profiles of *Porin*-Related Apoptosis Genes in Female Ticks

**FIGURE 7:**
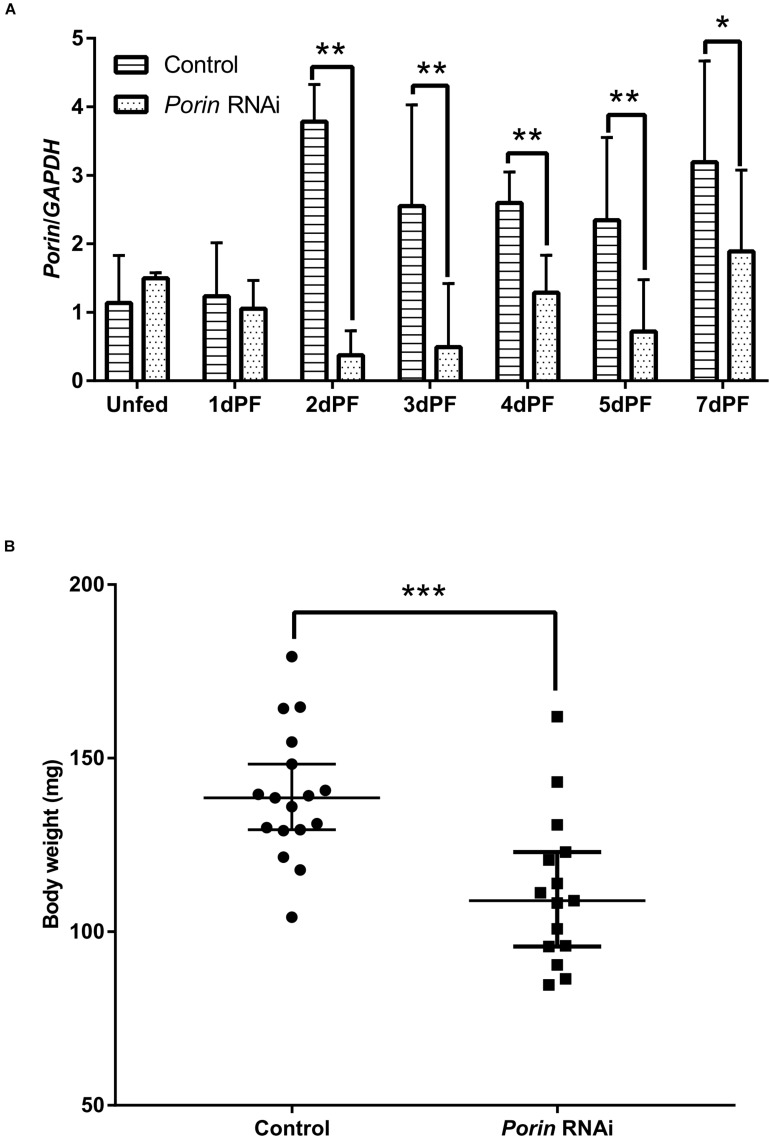
The effect of *porin* silencing on body weight of female ticks at engorgement. **(A)** Expression analysis of *porin* mRNA in whole body of dsRNA-injected female ticks; **(B)** Body weights of female ticks at engorgement in *porin* RNAi and control ticks. The bar indicates the median with 95% CI of three biological repeats. The asterisks above the bars indicate significant differences in *porin*/*GAPDH* and body weight between *porin* RNAi and control groups. *p < 0.05; **p < 0.01; ***p < 0.001.

**FIGURE 8:**
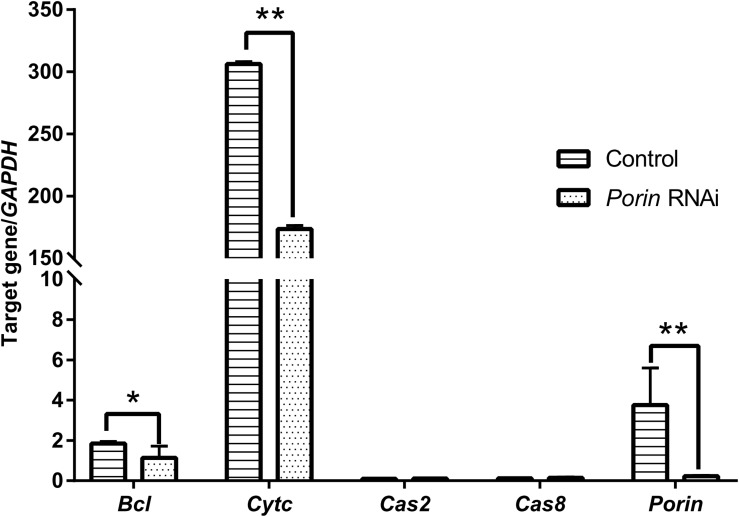
Impact of *porin* RNAi on *porin*-related apoptosis gene expression in engorged female ticks. The bar indicates the median with 95% CI of three biological repeats. The asterisks above the bars indicate significant differences in target gene/*GAPDH* between *porin* RNAi and control groups. *p < 0.05; **p < 0.01.

When each tick was injected with 1 μg of *porin* dsRNA, a gradual reduction in gene silencing efficiency was seen in the hamster-infested ticks after 2 days from tick attachment (Figure 7A). The gene was knocked down by 90.24% in female ticks fed on hamsters for 2 days (Figure 7A). The body weight of the engorged female ticks in the control group was significantly higher (*p* < 0.001) than that of the RNAi group (Figure 7B). No differences in feeding period were seen in the control and *porin*-knockdown groups (data not shown). The effect of *porin* silencing on *Babesia* burdens in the female ticks was time-course dependent. At 3dPF, 0 dAE, 1 dAE, and 8 dAE, the RNAi ticks had 2.34, 2.91, 2.16, and 4.26-fold lower *Babesia* burdens in comparison with the control ticks, respectively (Figure 5). However, at 4 dPF, 5 dPF, 3 dAE and 4 dAE, 2.04, 3.14, 2.82, and 2.41-fold higher amounts of *Babesia* DNA were detected in the *porin*-knockdown ticks, respectively (Figure 5). Furthermore, at 0 dAE the expression levels of *Cytc* and *Bcl* in the *porin*-knockdown female ticks significantly decreased in contrast to that in the control ticks (*p* < 0.01 and *p* < 0.05), whereas the mRNA levels of *Cas2* and *Cas8* did not show obvious changes in the *porin*-RNAi ticks compared with the control ticks (Figure 8).

## Discussion

*Porin* in *H. longicornis* is a 30.4 kDa protein with 282 amino acids, as is reported in other organisms (Sardiello et al., 2003; Wang et al., 2010; Rodríguez-Hernández et al., 2011). Additionally, our study showed that determinants of voltage gating and polypeptide binding sites in *porin* protein are conserved among tick species (Figure 2), suggesting that they play a primary role in the regulation of ion and molecular flow and in metabolism inside and outside the mitochondrial membrane among ticks. It was reported that *porin* might be involved in tick feeding and/or digestion of blood meals and its development (Ayllón et al., 2013; Rodríguez-Hernández et al., 2015). *Porin* mRNA levels of *I. scapularis* increased from egg to adult stages and from the non-feeding to feeding periods of female ticks. Knockdown of the gene resulted in about a 40% reduction in female tick weight after feeding compared to the weight of controls (Ayllón et al., 2013). *Porin* expression levels in the midgut of adult *R. microplus* ticks first increased to a maximum and then decreased at 0 to 72 h post repletion (Rodríguez-Hernández et al., 2015). In the present study, a similar expression level of *porin* mRNA was found in 1-day-laid and 7-day-laid eggs (Figure 3A), and the mRNA levels were appeared to be higher in the unfed nymphs, 12 hPF to 2 dPF nymphs, and the nymphs at 0 dAE than the nymphs at 2 dAE (Figure 3B). However, their expression levels increased in female ticks when taking blood from the hosts (Figure 7A). *Porin* silencing mediated by RNAi significantly decreased the body weight of engorged adults but did not alter the blood feeding period (Figure 7B).

When confronting stressful situations and adverse conditions, remodeling of the cell skeleton, inhibition of cell apoptosis, and manipulation of the innate or specific immune system can help hosts remove the damaged cells to maintain tissue homeostasis and therefore benefit the remaining cells (de la Fuente et al., 2016). In the regulated process of cell apoptosis, *porin* plays a pivotal role in releasing an apoptogenic factor, namely *Cytc*. Pathogen infection activates the Janus kinase/signal transducers and activators of transcription (JAK/STAT) to down-regulate *porin* expression and therefore inhibit cell apoptosis as an aid to pathogen infection, survival, development, and multiplication inside infected cells (Alberdi et al., 2015; de la Fuente et al., 2016). In the present study, *porin* mRNA expression levels appeared to be lower in *B. microti*-infected ticks than in uninfected ticks at engorgement when the highest *Babesia* burden occurred (Figures 5A,C,D), suggesting that the invasion of a large number of *Babesia* might inhibit cell apoptosis in ticks via suppression of *porin* expression. However, some other studies have reported opposite findings, showing the same or higher levels of *porin* expression in vectors when they have the highest pathogen load (Fongsaran et al., 2014; Rodríguez-Hernández et al., 2015; Jitobaom et al., 2016). These studies showed that *porin* may function as an activator of pathogen receptors (such as plasminogen) or a part of a pathogen receptor (such as *porin* plus *GRP78* complex). The formation of pathogen receptor facilitates pathogen entry into cells, for example, the dissemination of *Borrelia burgdorferi* in *Ixodes* ticks, *B. bigemina* in *Rhipicephalus* ticks, and the invasion of Japanese encephalitis virus, dengue virus, and *Plasmodium* spp. into the midgut cells of mosquitoes. Our data showed that nymphal and adult ticks acquired *B. microti* via blood sucking. During the blood feeding process, we found that the amount of babesial DNA in ticks increased at 4 dPF and 0 dAE, and then decreased thereafter, suggesting that *B. microti* infected and proliferated in the tick body at these timings. Moreover, *porin* mRNA expression levels appeared to be lower at 1 dPF, higher at 2–4 dPF, lower at 0–1 dAE, and higher at 2–3 dAE in the infected nymphs vs. the uninfected nymphs. The *porin* expression dynamics might be related to *Babesia* infection in a time-dependent manner. RNAi of *porin* changed the *Babesia* infection level in dsRNA-injected ticks in contrast to the control ticks. The peak of *Babesia* burden in control ticks was observed at 0 dAE, however, the peak in *porin* dsRNA-injected ticks was found at 4 dAE. Taken together, our results indicate that during the blood feeding *Babesia* infection might cause the inhibition of cell apoptosis at one time point and/or activation of *porin* expression for pathogen invasion at another time point (Alberdi et al., 2015; Rodríguez-Hernández et al., 2015; de la Fuente et al., 2016). For better understanding the interactions between *B. microti* and porin and the related molecules, further analyses will be needed focusing on an important organ for *Babesia* infection, such as the midgut.

Most *caspases* play a role in programmed cell death, including apoptosis and pyroptosis, and as initiators, executioners, or inflammatory types (Galluzzi et al., 2016). *Hlcaspase-2* (termed *Cas2* in our study) and *Hlcaspase-8* (termed *Cas8*) previously identified from *H. longicornis* (Tanaka et al., 2007) are two members of initiator *caspases* that might play important roles in inducing cell death by apoptosis. In addition to apoptosis, *Cas8* is required for the inhibition of necroptosis (Denecker et al., 2008). During times of cellular stress, mitochondrial *Cytc* binding to an adaptor protein (APAF-1) recruits initiator *caspases*, which helps to form a *caspase*-activating multiprotein complex called the apoptosome. Once activated, initiator *caspases* will modulate other executioner *caspases*. This leads to degradation of cellular components for apoptosis (Creagh, 2014). In our study, highly down-regulated *Cytc* induced by *porin* silencing did not suppress the expression levels of the initiator *caspase, Cas8* (Figure 8). The same phenomenon was observed in nymphs at 2 dAE and 2 dPF (Figure 3C), which might be explained by an extrinsic, and not intrinsic, apoptotic pathway available to *Cas8* (Creagh, 2014). Further experiments at protein level will be required to evaluate the role of *porin* in the activating *caspases*-interfered apoptotic pathway.

## Conclusion

In conclusion, the present experiments identified the *porin* gene from *H. longicornis* and evaluated its expression levels in *B. microti*-infected and -uninfected *H. longicornis* ticks at developmental stages. Our data suggest that *porin* might positively regulate expression of the *Cytc* gene, which is known to be vital for *caspases*-interfered cell apoptosis. *Porin* knockdown reduced body weight and changed *Babesia* infection levels in *H. longicornis* ticks. In addition, we detected DNA of the *B. microti* Gray strain in both nymphal and adult stages that fed on infected hamsters by using conventional and real-time PCR analyses. *Babesia* loads in nymphs and adults remained at low levels before engorgement, peaked at/around onset of engorgement, and then gradually decreased to low levels such as those in initial blood feeding stages, which is consistent with a previous observation of *B. microti* Munich strain infection in mice (Kusakisako et al., 2015). This *H. longicornis*-*B. microti* experimental infection model using hamsters will be used for further investigation of the interaction between ticks and human *Babesia*. Taken together, our findings will be useful for better understanding the roles of *H. longicornis porin* in tick development, blood feeding, and *B. microti* infection.

## Data Availability Statement

The datasets generated for this study can be found in the GenBank database under accession numbers MN584740 for porin and MN584741 for Bcl.

## Ethics Statement

The animal study was reviewed and approved by Animal Care and Use Committee of Obihiro University of Agriculture and Veterinary Medicine.

## Author Contributions

XX, RU-S, HS, QZ, and HC conceived and designed the study. WZ performed most of the experimental work. WZ and RU-S wrote the manuscript. WZ, KO, PAM, and ML collected and analyzed the data.

## Conflict of Interest

The authors declare that the research was conducted in the absence of any commercial or financial relationships that could be construed as a potential conflict of interest.

## Footnotes

1 https://blast.ncbi.nlm.nih.gov

2 https://www.ncbi.nlm.nih.gov/Structure/cdd/cddsrv.cgi

3 http://nadv.herokuapp.com/

4 http://www.bioinformatics.org/SMS/multi_align.html

5 https://www.bioinformatics.org/sms2/ident_sim.html

6 https://web.expasy.org/compute_pi/
