# Supplementary figures and images for: Porin Expression Profiles in Haemaphysalis longicornis Infected With Babesia microti
Source: Front Physiol. 2020 May 19;11:502. doi: 10.3389/fphys.2020.00502 (PMC7249857; doi:10.3389/fphys.2020.00502)

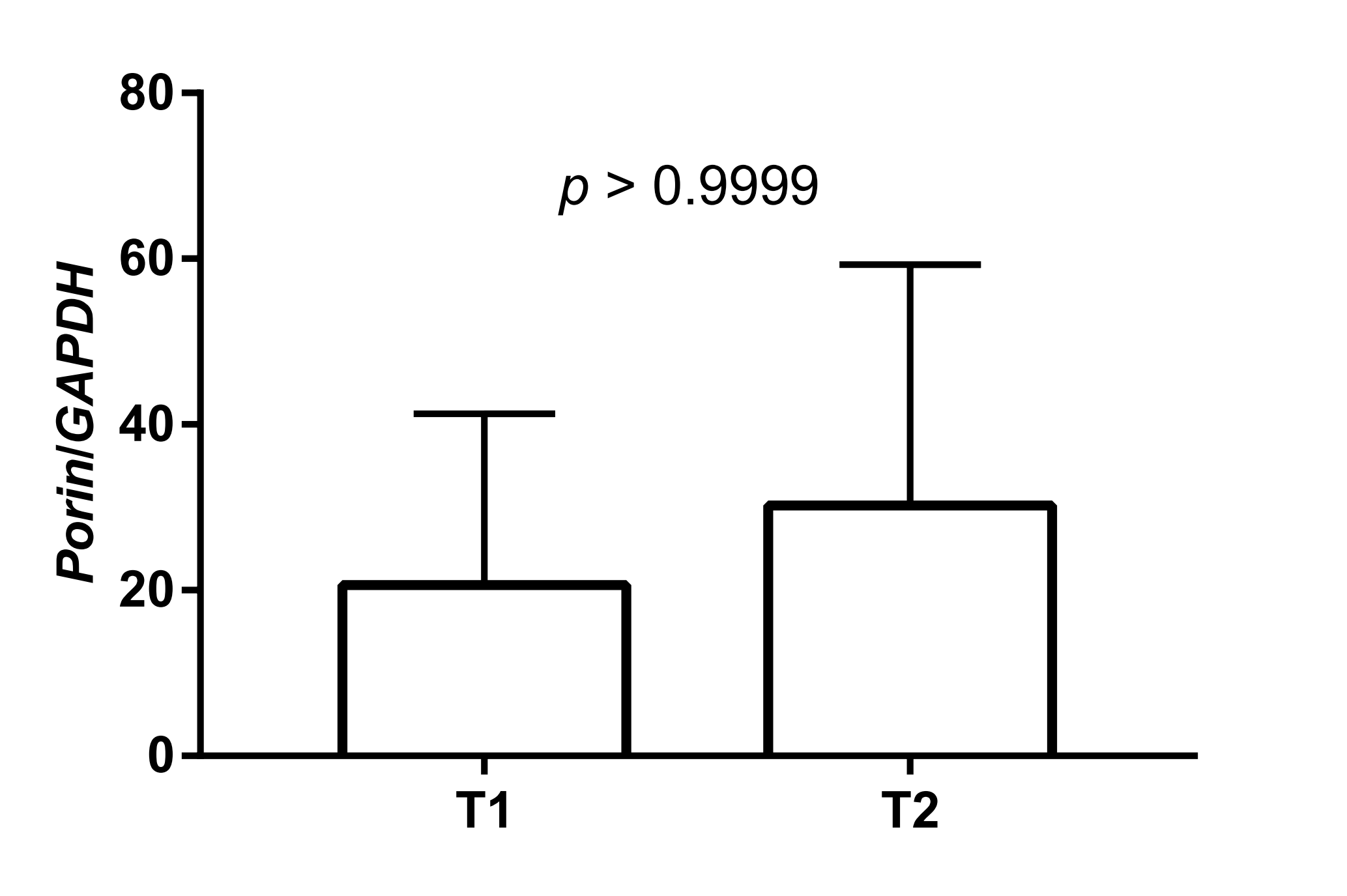

Supplement: Supplementary file 2 [file Image_2.TIF]
