# Supplementary material for: Porin Expression Profiles in Haemaphysalis longicornis Infected With Babesia microti
Source: Front Physiol. 2020 May 19;11:502. doi: 10.3389/fphys.2020.00502 (PMC7249857; doi:10.3389/fphys.2020.00502)
Supplement: Supplementary file 3 [file Table_1.docx]

Supplementary Materials

# Supplementary Table

| **SUPPLEMENTARY TABLE S1.** Sequences of primers used in this study. | | | | | |
| --- | --- | --- | --- | --- | --- |
| **Purpose** | **Gene** | **Upstream/downstream primer sequence 5′-3′** | **Ta/Et^¶^** | | **Reference** |
| Analyses of *Babesia* burdens in ticks | *Babesia β-tubulin* | ATGAGAGARATYGTACACATYCAAGC  TAYTGYTGGTAYTCGCTRACYA | 54°C/2 min | | (Hirata et al., 2011) |
|  | *Hlactin* | GCCCATCTACGAGGGTTACGCT  GTCGCGCACGATTTCACGCT | 61°C/45 s | | This study |
|  | *ITS-2* | TCCGCCGGTACTCCGGTTTCAG  CGTCAAAGAAACGTGCGCCCCC | 62°C/35 s | | This study |
|  | *Babesia* 18S rRNA | AAACGACTCCTTCAGCACC  ACCAGACAAATCACTCCACC | 52°C/35 s | | This study |
| Analyses of mRNA levels in ticks | *GAPDH* | TGTCCGTGGTAGACCTGACCTG  GGTCTTGGACAGAGCGATGCCA | 62°C/35 s | (Kawano et al., 2011) | |
|  | *L23* | CACACTCGTGTTCATCGTCC  ATGAGTGTGTTCACGTTGGC | 62°C/35 s | (Umemiya-Shirafuji et al., 2014) | |
|  | *HlP0* | CTCCATTGTCAACGGTCTCA  TCAGCCTCCTTGAAGGTGAT | 62°C/35 s | (Umemiya-Shirafuji et al., 2014) | |
|  | *Hlactin* | GCCCATCTACGAGGGTTACGCT  GTCGCGCACGATTTCACGCT | 62°C/35 s | This study | |
|  | *Porin* | CGTGAAGCTCGACTGCAAGTCC  GCGAGTGTGTTGTCCGTGTTCC | 62°C/35 s | This study | |
|  | *Bcl* | AGTCGTCGTGTTGTGTTG  GTTCACCATCTCGTTCACC | 58°C/35 s | This study | |
|  | *Cytc* | TCTCAAGAGACATTTCAACTGC  CACCTCAAAATGACATTTGTCC | 60°C/35 s | This study | |
|  | *Cas2* | ACCAAAGAGCCAAAAGCC  TGACAATGATACAACTACCACG | 58°C/35 s | This study | |
|  | *Cas8* | CAATACGAGACGCACACAC  TAGCGATTCCTTTTCCGCC | 58°C/35 s | This study | |
| ^¶^Ta, annealing temperature; Et, extension time | | | | | |

**References**

Hirata, H., Kawai, S., Maeda, M., Jinnai, M., Fujisawa, K., Katakai, Y., et al. (2011). Identification and phylogenetic analysis of Japanese Macaque *Babesia*-1 (JM-1) detected from a Japanese Macaque (*Macaca fuscata fuscata*). *Am. J .Trop. Med. Hyg*. 85, 635–638. doi: 10.4269/ajtmh.2011.11-0035

Kawano, S., Umemiya-Shirafuji, R., Boldbaatar, D., Matsuoka, K., Tanaka, T., and Fujisaki, K. (2011). Cloning and characterization of the autophagy-related gene 6 from the hard tick, *Haemaphysalis longicornis*. *Parasitol. Res*. 109, 1341–1349. doi: 10.1007/s00436-011-2429-x

Umemiya-Shirafuji, R., Galay, R. L., Maeda, H., Kawano, S., Tanaka, T., Fukumoto, S., et al. (2014). Expression analysis of autophagy-related genes in the hard tick *Haemaphysalis longicornis*. *Vet. Parasitol*. 201, 169–175. doi: 10.1016/j.vetpar.2014.01.024

# Supplementary Figures

**SUPPLEMENTARY FIGURE S1.** The mRNA expression levels of several candidate internal control genes. The mRNA expression levels of internal control genes were examined in the infected and non-infected nymphs at engorgement, and in the 2-d-fed female adults without *Babesia* infection.

**SUPPLEMENTARY FIGURE S2.** *Porin* gene expression levels in unfed nymphs cultured at 15°C and 25°C. T1, unfed ticks cultured at 15°C; T2 unfed ticks cultured at 25°C. The bar indicates the median with 95% CI of three biological repeats.
